# Sex, size and habitat complexity effects on emergence latency and latency to locate food of the invasive porthole livebearer *(Poeciliopsis gracilis)*

**DOI:** 10.1371/journal.pone.0269384

**Published:** 2022-06-09

**Authors:** Esteban Aceves-Fonseca, Abigail Santiago-Arellano, Morelia Camacho-Cervantes

**Affiliations:** Laboratorio de Ecología de Especies Invasoras, Instituto de Ciencias del Mar y Limnología, Universidad Nacional Autónoma de México (UNAM), Coyoacán, Ciudad de México, México; University of Florida, UNITED STATES

## Abstract

Biological invasions are considered the second major cause of plant, amphibian, reptile, and mammal loss worldwide. Like islands, freshwater ecosystems are especially susceptible to the negative impacts of invasions. The porthole livebearer (*Poeciliopsis gracilis*), recently identified as invasive in the Mexican Central Plateau, is increasing its populations and could impact freshwater ecosystems like its cousin species the guppy (*Poecilia reticulata*). Risk-taking behaviours, such as emergence latency, are recognised as key characteristics to invasion success and Poeciliid females can establish a viable population by themselves (due to their multiple paternity broods). We investigated the emergence latency and latency to locate food in simple and complex environments of porthole livebearers, including the effect of their size and sex. For both sexes, bigger fish emerge less times and take longer to do so, but females are faster to exit the refuge than males. We found no differences in porthole livebearer’s behaviour in complex or simple habitats, and no significant differences between sex, size or treatment in the time to locate food after exiting the refuge. Our results suggest that the benefit of faster emergence from the refuge in porthole livebearers in novel environments could be higher for females. We consider that porthole livebearer females being bolder could contribute to the invasion success of the species. Our study points at females and smaller fish as being the more likely to explore novel environments, which could contribute to understanding how the invasions by the porthole livebearer are driven.

## Introduction

Invasive species are the second major cause of plant amphibian, reptile, bird and mammal extinctions surpassed only by habitat loss [1]. Impacts on ecosystems can cost billions of dollars due to the efforts for containing and preventing biological invasions [2–4]. Invasions of freshwater ecosystems are of special concern since, like islands, the available area to be used by its inhabitants is delimited by space that is unsuitable [5–7].

Invasive freshwater fishes are considered especially dangerous due to their negative impacts on the ecosystems they invade. Considering that freshwater is a valuable resource for the different forms of life on the planet, it is important to investigate the aspects promoting invasions by fishes, and their negative impacts [8, 9]. For example, brown trout *(Salmo trutta)* was introduced in streams of New Zealand causing multilevel impacts. They changed completely the composition of the algae in the area, and at the ecosystem level the entire annual population of invertebrates were consumed by trout [10].

Studying the mechanisms that promote or decrease the fitness of exotic individuals at each stage is crucial when it comes to predicting the success or failure of inadvertent species’ introduction [11, 12]. Over the past two decades, animal behaviour has gained special attention in explaining the invasion process and behaviour plays an important role in each stage of an invasion (*i*.*e*., movement, introduction, establishment, and propagation) [11, 13, 14]. Invasive species share some behavioural and life history traits that are associated with greater invasion success, such as high fertility, high plasticity and tolerance to changing temperatures, high levels of interspecific aggression, and innovative foraging or high exploratory activity [11, 15, 16]. An example of high plasticity is observed in cane toads, introduced to Australia, where some populations are changing their feeding habits from nocturnal to diurnal due to intense intraspecific competition for food [18]. A relevant characteristic of animal behaviour for the success of their invasion is the tendency towards risk-taking behaviours; indeed, individuals that emerge faster from a refuge are more likely to explore novel environments or approach humans, increasing their likelihood of being transported to new areas [17]. Risk-taking behaviour tendency is driven by the need to disperse and explore novel environments that could lead to the opportunity of finding food, new shelters, partners, etc. [18].

Several studies have focused on and recognised boldness implications for the success of biological invasions in animals [19–21], and fish in particular [16–18, 20, 21]. An important family of invasive freshwater fish throughout the world and recognised as bold is the Poeciliidae [22]. In Mexico, the Poeciliidae represents the second major invasive family, surpassed by the Cyprinidae family [23]. The negative impacts of Poeciliid invasions have been recognised as contributing to the endangerment of native endemic species in the Mexican Central Plateau [24–28]. The endangered Goodeidae family, which comprises *ca*. 49 species, is one of the families affected by sharing habitat with poeciliids [29, 30].

The Poeciliidae family is composed of *ca*. 300 species of viviparous fishes distributed from the Mississippi river to Argentina [31–34]. The majority of Poeciliid fishes are native to Central America (from the South of the USA to Argentina), some of them were purposefully or accidentally translocated between regions, and are now considered invasive freshwater fishes in some areas of the Americas and around the globe [35–37]. Such is the case of the porthole livebearer (*Poeciliospsis gracilis*), a fish native to the East basin of Mexico. This fish has been used as bait or as ornamental fish for amateurs aquarists, and as a biocontrol for mosquitoes in El Salvador [38–40]. The porthole livebearer has been introduced accidentally in the Mexican West and Central basins, where it is now considered an invasive species [41–44]. Indeed, a 2012 study of invasive fishes in rivers at the Mexican *Reserva de la Biósfera Sierra Huautla*, revealed that porthole livebearers had the highest increase of relative abundance since 1994 compared to the other species of fish present in the area [45]. Another study in rivers at the *Chontalcoatla-Amacuzac* hydrologic system in the western Mexico Basin found that this species was the most abundant, representing 48% of the total fish in the area (native and invasive) and 64% of the total invasive fish [46]. However, little is known about the mechanisms of invasion of the porthole livebearer, including their biology and species-specific behaviours. Although the study of animal behaviour is a relatively new approach, its importance is increasingly being recognised in explaining and predicting the success of invaders and, therefore, contributing to risk management/assessment to develop management plans and identify possible invaders [47].

Here we investigated the emergence latency and latency to find food of male and female porthole livebearers in complex and simple habitats. Complex microhabitats can be important to some fish species because they provide a refuge against predators [48–51]. For example, adult guppies engage faster in exploratory behaviour when the habitat is more complex [52]. This could be because complex environments provide shelter and protection from predators [53]. However, this might not be the case for young fish, who might have different risk perceptions as they are smaller and less experienced, and thus have a different refuge use behaviour. We then hypothesise that complex environments will act as promoters for risk-taking behaviour and reduce the emergence latency of porthole livebearers. Regarding sex, it is known that in other species of poeciliids males are more prone to risk-taking behaviours than females [54, 55]. We then expect male porthole livebearers in our experiment to emerge from the refuge faster than females and, therefore, find food faster.

In Mexico, this species inhabits shallow ponds and riverbanks that are mostly open, leaving the sides as shelters and few rocks or plants in the middle as hiding places (MCC Pers. Obs). We believe this information on risk taking behaviours could contribute to assessing the potential success of porthole livebearers as invaders.

## Material and methods

Porthole livebearer fish used in this experiment were acquired in October 2019 at the Mixihuca Fish Market in Mexico City; fish in the market are originally from the state of Morelos but bred in the market to sell as bait for fishing and food for larger fish. The fish were transported to the aquarium at the Institute of Marine Sciences and Limnology, UNAM. Adults were selected, and their taxonomic identity verified according to Miller et al., 2005; males were easily identified as their anal fins are modified into a gonopodium where females are not. Forty-six fish were placed in a quarantine tank (50 cm x 30 cm x 26 cm) filled with aged tap water. Tanks were conditioned with bottom gravel and a preventive anti-parasite treatment consisting of malachite green, methylene blue and aquarium salt, also we added zeolite to control ammonia levels in the water. Each tank contained a filter, plastic plants and a Lomas AquaFlow10F® aerator for oxygen supply.

**Fig 1:**
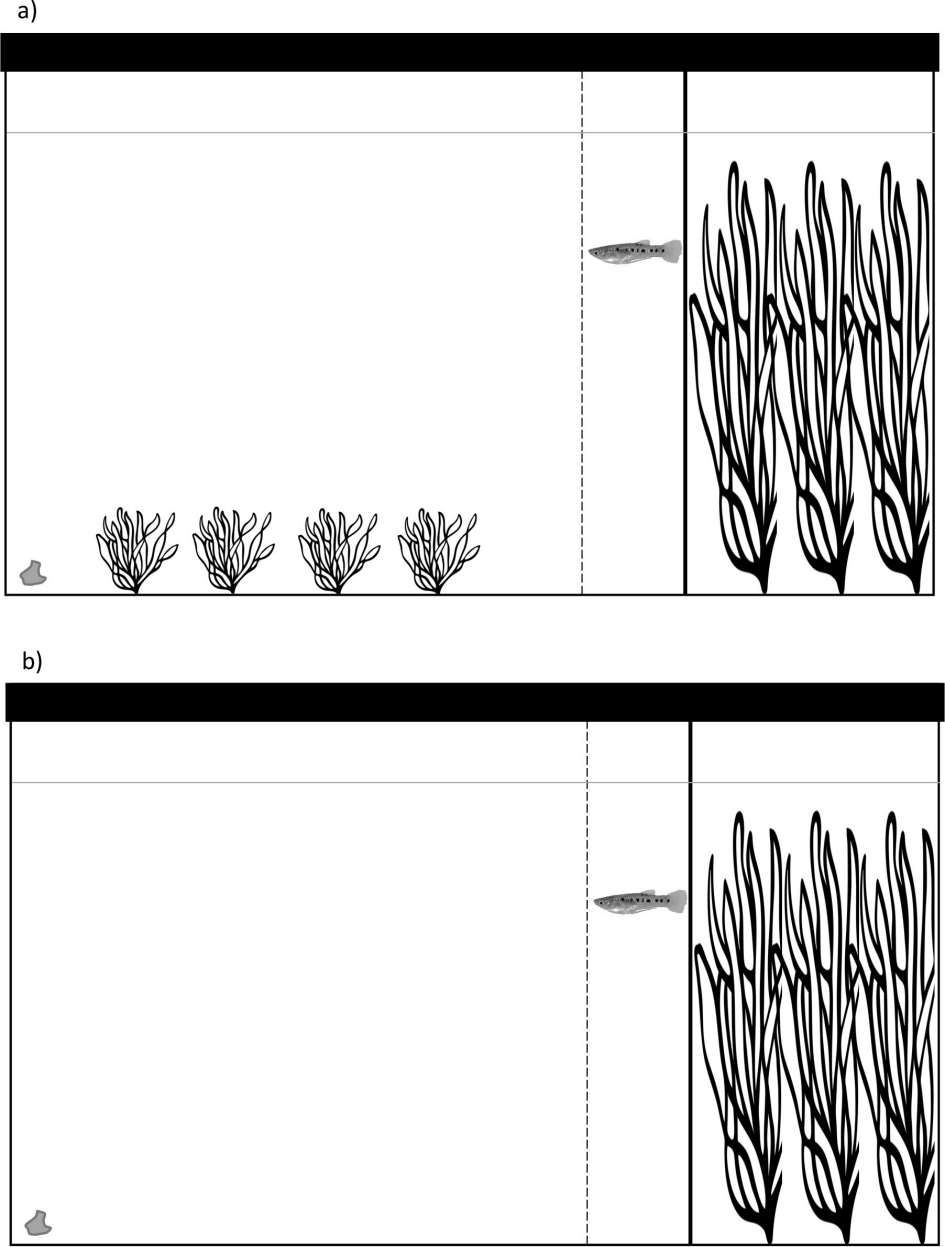
a) Diagram of the complex habitat: this habitat had a refuge built with 20cm tall plants (right), and 4cm tall plastic plants outside of the refuge (left). Two lines marked in the crystal designated the “refuge line” (a solid line, adjacent to the refuge) and the “starting line” (towards the 4cm plants). A food flake was submerged on the far end of the tank from the refuge (far left). b) Diagram of the simple habitat: this habitat had only a refuge (right) built with 20 cm tall plants, two lines marked in the crystal designated the “refuge line” (a solid line, adjacent to the refuge) and the “starting line” between the refuge line and the open area), and a food flake submerged (far left).

Fish were fed twice a day with commercial flakes and photoperiod was twelve hours of light and twelve hours of darkness. After quarantine time (20 days) was over, fish were fed only once at 14:00 h to ensure fish to be observed the following day had a starving period of at least 20 hr. Focal fish to be observed were selected randomly from stock tanks and carefully transported in a 1 L container to the observation room. The experiment was conducted over 12 consecutive days in the observation room inside the aquarium. We set up an observational tank (50 cm x 30 cm x 26 cm) where we simulated the two habitats: simple and complex. We alternated the days between simple and complex habitat treatments to avoid biases. Both habitats contained gravel bottom, and refuge built with eight plastic plants of 20 cm height. The simple habitat only had eight plastic plants, while the complex habitat contained four additional small plastic plants distributed in the area outside of the eight-plant refuge (Fig 1). The observation tank was marked with two lines (visible to the observer but not to the fish): (1) the “refuge line” indicating the limit of the refuge, and (2) the “starting line” that established the point at which we considered the fish left the refuge (Fig 1). The distance between one line and the other was 2.5 cm, which is approximately a porthole livebearer’s body length–the average size of all fish used in this experiment is 2.38 cm. All fish were observed only once and discarded to a different stock tank to avoid confusion; thus, all our observations are independent from each other. Right before each observation, a flake of approximate 2 cm^2^ was submerged in the opposite extreme of the refuge in the observation tank, at the end of the observation any flake remaining was removed. In total 60 fish (30 males and 30 females) were observed, one focal fish at a time. For the complex environment 15 females and 15 males were observed and for the simple environment 15 females and 15 males. Observations started when the fish were gently released by submerging a small container (approximately 200 ml) into a corner of the tank in the refuge side and allowing the fish to transfer into the observation tank, in all cases fish swam into the refuge without signs of stress. We recorded two variables: 1) emergence latency–seconds it took focal fish since entering the refuge until crossing the starting line with its entire body length, and 2) latency to locate food–seconds it took focal fish to locate food since surpassing the starting line (database of recordings in S1 Data). After being observed, all fish were photographed and their standard length was measured using the ImageJ® software [56]. Female fish in our experiment had an average standard body length of 2.3 cm (SD = 0.51 cm), while males’ average was 2.38 cm (SD = 0.53 cm). Fish that were observed in the complex habitat treatment had an average standard body length of 2.62 cm (SD = 0.4 cm), and those observed in the simple habitat treatment had an average of 2.14 cm (SD = 0.51 cm).

### Ethical note

Experiments for the purposes of this article were conducted at the Universidad Nacional Autónoma de México in Mexico City using fish of only one species (*Poeciliopsis gracilis* ~60 individuals). The study involved behavioural observations in glass aquarium tanks, which did not include any surgery, anaesthesia, or other invasive procedure that could have produce distress in the fish. Mortality was negligible (<5%) and once the experiments were completed, fish were returned to the aquariums to be kept for future research projects. All methods above were revised and approved by *Subcomité de Bioética de la Comisión de Ética Académica y Responsabilidad Científica de la Facultad de Ciencias*, UNAM with the folio: T_2019_02_026; and are in accordance with the Guide for the Care and Use of Laboratory Animals [57]. Fish were transported to the laboratory following the Official Mexican Norm NOM-051-ZOO-1995 for humanitarian treatment in the mobilization of animals. Laboratory protocols followed all guidelines provided by the Mexican Official Norm NOM-062-ZOO-1999 for the use and maintenance of vertebrates for research purposes.

### Statistical analysis

We performed a Generalized Linear Model (glm) to test for the effects of sex, size and habitat complexity in emergence latency and latency to locate food. Since neither emergence latency nor latency to locate food were normally distributed, we specified a Gamma distribution (after obtaining a p>0.05 in the Kolmogorov-Smirnov test for Gamma distributions for both variables). We ran our two models, for emergence latency and latency to locate food, including the interactions between sex, size and habitat complexity, but since none of the interactions in either of the models were significant, we removed interactions from the analysis to increase clarity. We included linear model tendency lines in our graphs to improve visualisation of our data, since it distributes Gamma and the tendency lines from our glms were flattened at the bottom of the vertical axis. Statistical analyses and graphs were performed using RStudio 2021 [58].

## Results

### Emergence latency

**Fig 2:**
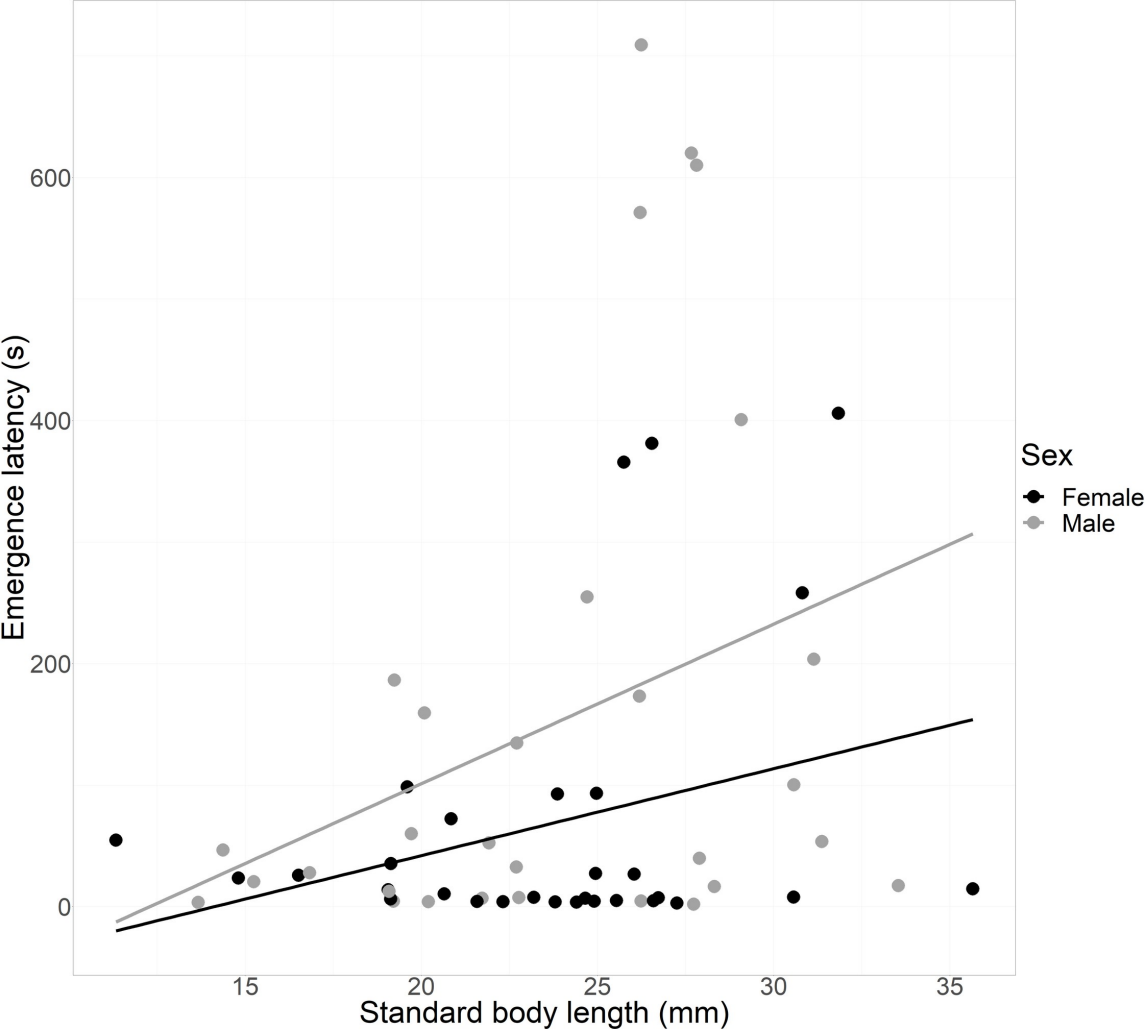
Emergence latency. Females were faster than males to leave the refuge. Tendency lines are for visualisation of tendencies and derive from linear models for females (y = -100.97 + 7.14 x, rˆ2 = 0.062) and males (y = -161.4 + 13.12 x, rˆ2 = 0.07).

Females were faster to exit the refuge than males (glm: t_2,60_ = 2.46, p = 0.035, y = 2.214 + 0.831 x, rˆ2 = 0.08, Fig 2), and, for both males and females, fish with smaller size took less time to exit the refuge than larger fish (glm: t_2,60_ = 2.33, p = 0.023, y = 2.214 + 0.831 x, rˆ2 = 0.08, Fig 2). Fish behaviour did not differ significantly between habitat complexity treatments (glm: t_2,60_ = 1.34, p = 0.18, y = 2.214 + 0.831 x, rˆ2 = 0.08).

### Latency to locate food

**Fig 3:**
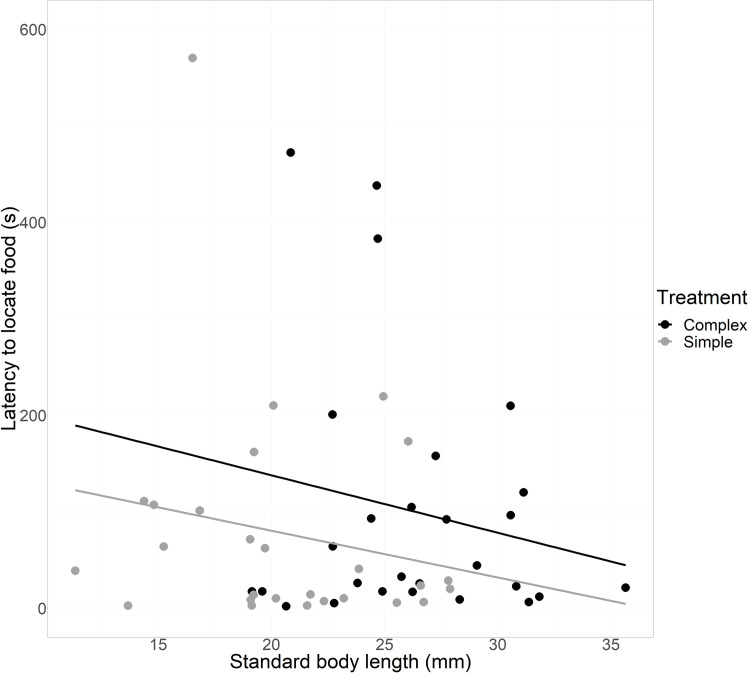
Effect of treatment on latency to locate food. Tendency lines are for visualisation of tendencies and derive from linear models for complex habitat (y = -124.23 + 10.38 x, rˆ2 = -0.004) and simple habitat (y = -93.118 + 7.73 x, rˆ2 = -0.003).

Males and females showed a similar latency to find the food after exiting the refuge (glm: t_2,60_ = 0.7, p = 0.49, y = 6.713–0.246 x, rˆ2 = 0.04, Fig 3). There were no differences between fish sizes (glm: t_2,60_ = 1.925, p = 0.06, y = 6.713–0.246 x, rˆ2 = 0.04, Fig 3) or habitat complexity treatments (glm: t_2,60_ = 1.749, p = 0.086, y = 6.713–0.246 x, rˆ2 = 0.04, Fig 3) in fish’ latency to locate food.

## Discussion

Sex and size of fish were the most influential variables for the exit emergence latency. We found smaller fish have a shorter emergence latency regardless of their sex or the complexity of the environment, but in general males were slower to emerge than females. We found no differences in focal’s latency to locate food between sexes, sizes or habitat complexity in the environment. Contrary to our hypothesis, habitat complexity was not a variable affecting porthole livebearers in this study, and females showed a higher tendency to risk-taking behaviour. The r^2 values for both our models were low (0.08 and 0.04), which means the variability in porthole livebearers’ behaviour explained by our independent variables (sex, size and treatment) is low. Still, we consider our results are of value to understand the behaviour of porthole livebearers, since this experiment was fully controlled on its environmental conditions, our p values were significant, and our sample size was not bigger than other sample sizes used in similar experiments. We then hypothesise this variability is due to the variability in the individual behaviour of porthole livebearers, which is also a valuable characteristic for species to become invasive [59]. However, this idea should be further researched.

We found females have a shorter emergence latency than males, which also is contrary to our hypothesis given that Poeciliid males have been found to have a higher tendency to risk-taking behaviours [54, 55]. This is also the case for the invasive fish *Neogobius melanostomus* and *Brachyrhaphis spp*, for which males are also bolder than females [60, 61]. Boldness is key trait affecting dispersal, which in turn is key for invasive species success [18]. A review by Li & Kokko (2019) [62] argues that dispersion is sex-biased when there is a differentiated competition for resources and a motivation to avoid inbreeding between males and females. Our results are in line with Li & Kokko (2019) [62] findings regarding females benefiting more from dispersing as it could enable them to locate resources while avoiding male’s harassment, which is an important source of reduction for female Poeciliids’ fitness [63]. However, further research should be made, as if this were the case, guppy females would be expected to be bolder too. The fact that porthole livebearer females are more prompt to abandon a refuge could represent a key characteristic in their invasive process, especially at the beginning, as in many Poeciliid species adult females could store sperm from multiple males [64–66], and for guppies this is a characteristic that has enabled even a single female to form a viable population [35]. We thus hypothesise that the invasion of porthole livebearers could be female-biased. Still, we acknowledge that the variability of our data is high, our adjusted r^2 for our models were low even if our p values were significant.

Previous studies have found that size has an effect on fish tendency to engage in risky behaviour; but, as with sex, it might be a species-specific trait. While some authors have found that bigger fish, no matter the sex, take more time to leave a refuge than smaller fish, some studies correlate bigger body sizes with shorter emergence latencies, and other studies find no relation between size and emergence latency [67–70]. A possible explanation for smaller porthole livebearer’s shorter emergence latency might be that smaller animals have a higher specific metabolic rate, which translates into greater nutritional requirements and a bigger motivation to engage in searching activities outside of refuges [71]. And it is also possible that younger, and smaller, individuals decide to take bigger risks due to their lack of experience [67]. Nevertheless, this explanation would need further research as fish used in our experiment were captive bred, which would reduce the variability of scenarios our individuals have faced even if older. Our focal fish reflect the behaviour of fish in captivity; however, these fish are most likely the ones that would end up in the wild starting exotic populations.

Environment complexity showed no significant effect on emergence latency for porthole livebearers, while similar differences have proved significant for other poecilids [52]. Contrary to our results, in the case of the guppy, authors found distinct emergence latencies depending on the complexity of the environment [52], so the results of the present experiment may simply suggest that the complexity of the ambient is not a relevant factor for the exploratory behaviour of porthole livebearers. These results also suggest that particular characteristics of individuals, such as size and sex, can play a more important role than the environment. However, this needs to be further studied for the species to be able to make a final conclusion.

Regarding the time it took fish to locate food once out of the refuge, we found no differences between sexes, sizes or habitat complexities. Still, size variable was very close to being significant (p = 0.06) which might suggest that there could be an effect of size (larger fish locate food faster) but it is not strong enough to be found with our sample size. Based on our results, we hypothesise that large porthole live bearers might take longer to leave a refuge but could be faster to locate food once engaged in searching behaviour. Since our results were not conclusive for this, but they show a trend, we think a specific experiment and larger sample size could bring more insights in the matter.

In the specific case of females, other studies have found that when female fish perceive more risks in the environment (e.g., predator signals), bigger individuals are faster at emerging from a refuge while smaller fish display shy behaviours [72]. Our results are consistent with what was reported by Olivera et al., (2017) [67], who examined the relationship between size and risk-taking behaviour in eight Poeciliid *Brachyraphis episcopi* populations, and found that larger fish took longer to leave the refuge. However, this is not the case for all Poeciliid species. Harris et al., (2010) [54] found that the variables that influence the emergence latency of guppies are mainly sex and predation risk in their locations of origin, while size was irrelevant. This is also the case for *Brachyrhaphis roseni* and *Brachyrhaphis terrabensis*, Brown C. et al., (2004) [60] found that size had no effect in the tendency to take risk of fish, but contrary to our results, they found that males are more willing to take risks than females.

In the same species it is possible to find distinct dispersion capacities in accordance to the sex, in such way that the individuals of the more dispersive sex can promote the establishment and adaptation of the population in new environments and territories [73]. For porthole livebearers, the fact that females seem to be bolder than males potentially makes the dispersion of the species more efficient than that of those species that are male dispersed. Further studies should explore how porthole livebearers behave in distinct contexts and take into account the conditions of the localities from which the populations to be observed came from.

## Conclusion

In this study, we observed how porthole livebearer individuals behave when they face the possibility to explore a new environment, one of the first stages of invasion that represents a challenge that all invasive species have to deal with. Larger fish took more time to leave the refuge and females abandoned the refuge sooner than males. These differences can be explained by intrinsic characteristics of each sex. In poeciliids it has been recorded that females engage more on searching for food and dispersing than males, who engage more than females in other activities like reproductive behaviour [74]. Males then would be expected to leave the refuge in search of mates, which they did but took longer than females, possibly because there were no other fish in the tank while there was food. Other studies have found that reproductive females need to feed more, which allows them to have a greater reproductive capacity and at the same time become larger individuals [52, 72]. These results are in accordance with the idea that emergence latency and activities in which fish invest their energy change as they develop [75]. It is possible that the invasions by porthole livebearer are female-biased. In this experiment we used fish from a fish market, which are likely to end up in the wild after being discarded from home aquaria and as bait for larger fish thus the behaviours of these porthole livebearers potentially will impact native fish communities.

## Supporting information

S1 Data (R)

S2 Data (TXT)
